# Longitudinal Associations between Obesity, Inflammation, and the Incidence of Type 2 Diabetes Mellitus among US Black and White Adults in the CARDIA Study

**DOI:** 10.1155/2020/2767393

**Published:** 2020-08-15

**Authors:** Sharon H. Jackson, Anna Bellatorre, Timothy McNeel, Anna María Nápoles, Kelvin Choi

**Affiliations:** ^1^Division of Intramural Research, National Institute on Minority Health and Health Disparities, 9000 Rockville Pike, Building 3, Floor 5, Room 5W13, Bethesda, MD 20892, USA; ^2^LEAD Center, Colorado School of Public Health, Anschutz Medical Campus, 12474 E 19th Avenue, Rm. 112, Campus Box F426, Aurora, Colorado 80045, USA; ^3^Information Management Services, Inc., 3901 Calverton Blvd., Suite 200, Calverton, Maryland, USA; ^4^Division of Intramural Research, National Institute on Minority Health and Health Disparities, 9000 Rockville Pike, Building 3, Floor 5, Room E08, Bethesda, MD 20892, USA; ^5^Division of Intramural Research, National Institute on Minority Health and Health Disparities, 9000 Rockville Pike, Building 3, Floor 5, Room 5W05, Bethesda, Maryland 20892, USA

## Abstract

**Aim:**

Assess prospective relationships between obesity and inflammation on the incidence of type 2 diabetes mellitus (T2DM).

**Methods:**

A cohort of nondiabetic respondents from the Coronary Artery Risk Development in Young Adults (CARDIA) study was followed from 2005-2006 (wave 7) to 2010-2011 (wave 8). Diabetes status was determined in wave 8 based on self-report, blood glucose level, and anti-hyperglycemic medication use in conjunction with a homeostatic model assessment-based classification for distinguishing diabetes subtype. We performed a series of multivariable logistic regression analyses to assess the relative influence of obesity (waist circumference) and individual inflammatory biomarkers (i.e., C-reactive protein, fibrinogen, and sex-specific serum uric acid and gamma-glutamyltransferase) on the odds of developing incident T2DM between waves 7 and 8.

**Results:**

Among 2784 nondiabetic CARDIA respondents, 146 (5.2%) new cases of T2DM were identified between waves. Having a high waist circumference (AOR = 6.15; 95%CI = 4.14, 9.14) and being Black (vs. White) (AOR = 1.60; 95%CI = 1.05, 2.44) were associated with T2DM. Adjusting for inflammation biomarkers attenuated the effects of waist circumference and race with T2DM. Clinically elevated CRP (AOR = 1.83; 95%CI = 1.18, 2.82) and uric acid (AOR = 2.57; 95%CI = 1.70, 3.89) predicted T2DM among all respondents. However, stratification by race showed greater attenuation of the effects of waist circumference on T2DM in Whites than in Blacks when inflammation biomarkers were accounted for in the model.

**Conclusion:**

Targeted control of systemic inflammation may reduce the risk of developing T2DM, especially among Blacks, and could help address Black-White disparities in diabetes care and outcomes.

## 1. Introduction

Type 2 diabetes (T2DM) is the most common type of diabetes in adults. It is estimated that 90% of all cases of diabetes in adults are due to T2DM [1]. Risk factors for T2DM include being overweight or obese, physical inactivity, increased age, minority race or ethnicity, having a first-degree relative with diabetes, and the presence of clinical risk factors including having a history of cardiovascular disease, hypertension, elevated cholesterol and/or triglycerides, and low HDL cholesterol [2]. Notable among these risk factors is the global obesity epidemic, which is contributing to an increasing incidence of T2DM worldwide in older adults, and now in children and adolescents as well. Obesity negatively impacts most physiologic systems, increasing the risk for cardiometabolic morbidity, such as impaired glucose metabolism, dyslipidemia, renal and cardiovascular disease, and hypertension [3–5].

Chronic inflammation occurs as a consequence of obesity, and obesity-associated inflammation is both systemic and organ specific [3, 4]. Obesity-associated inflammation has been implicated in cardiometabolic disease, and investigations to discern the complex pathways linking obesity, inflammation, and T2DM are ongoing [4, 5]. The associations of many clinical inflammatory biomarkers with obesity and T2DM risk remain unclear.

Inflammatory biomarkers that are thought to be associated with obesity include C-reactive protein (CRP), fibrinogen, uric acid, and gamma-glutamyltransferase (GGT) [6–13]. CRP is an acute-phase reactant that is produced in the liver largely in response to interleukin-6 (IL-6) stimulation [11]. CRP production is a nonspecific marker of systemic inflammation. Blood levels of conventional CRP are used clinically to detect acute inflammation typically following an injury or during an infection [7]. In contrast, the measurement range for high-sensitivity CRP extends below the range for conventional CRP, which allows for its use as a biomarker of low-grade systemic inflammation in otherwise healthy appearing individuals [7]. As such, high-sensitivity CRP is one of the most commonly used inflammatory markers for evaluating cardiometabolic disease risk in epidemiological research. Fibrinogen is a critical factor in the complex cross-talk between coagulation and inflammation, both of which are activated in response to vascular insult, tissue injury, or infection [14]. As a proinflammatory intermediary, fibrinogen binds to and activates immune cells. Similar to CRP, fibrinogen is an acute-phase reactant that is produced in the liver largely in response to proinflammatory cytokine stimulation. Prior studies examining the relationship between visceral fat and inflammation reported that African Americans and Hispanics had higher concentrations of the acute-phase reactants CRP and fibrinogen as well as IL-6 than Whites, but lower amounts of visceral adipose tissue compared to Whites [13]. Uric acid is generated in the liver following the breakdown of purines that are endogenously produced or consumed from purine-rich dietary products (e.g., red meat, beer, liquor, and high fructose corn syrup). Hyperuricemia occurs when the body produces too much uric acid or when the body fails to excrete excess uric acid. GGT is a glycoprotein found on the plasma membrane of most cells and organs; it is most abundant in hepatocytes and plays a primary role in glutathione metabolism [9]. Clinically, GGT is primarily used to assess liver function, including hepatic inflammation with or without overt hepatitis [9].

In this study, we sought to test prospectively whether obesity and inflammatory biomarkers assumed to be obesity related are associated with the risk of developing T2DM among adults, and whether controlling for inflammatory biomarkers attenuates the association between obesity and T2DM. We also sought to determine if any observed associations between inflammatory biomarkers and T2DM risk varied by race. Identification of useful inflammatory biomarkers for assessing clinical T2DM risk could assist efforts to prevent T2DM and reduce racial disparities in obesity and T2DM.

## 2. Materials and Methods

### 2.1. Sample

Data for these analyses come from the Coronary Artery Risk Development in Young Adults (CARDIA) study [15]. The CARDIA study was designed as a prospective longitudinal study to evaluate the risk of developing heart disease over time for Black and White adults from 4 regions in the United States: Birmingham, AL; Chicago, IL; Minneapolis, MN; and Oakland, CA [15, 16]. A detailed description of recruitment methods and participant sampling have been documented elsewhere [16]. Institutional review boards at the participating study sites (University of Alabama at Birmingham, University of Minnesota, Northwestern University, and Kaiser Permanente Oakland, California) gave approval for the study, and study participants provided written informed consent prior to enrollment. This research only involved the use of deidentified data, which is not considered human subject research and requires no Institutional Review Board review or approval per National Institutes of Health policy and 45 CFR 46.

CARDIA participants were selected to balance the number of Black and White participants in subgroups for sex, education (high school or less and more than high school), and age [16]. The CARDIA study also has collected biological and survey data related to health and social context. The CARDIA study began in 1985-1986 with approximately 5,115 adults aged 18-30 years enrolled at the start of the study. A majority of the study participants have been evaluated at varying intervals ranging from 1 to 5 years in several subsequent follow-up examinations through 2016 [15]. The data we used come from examination waves 7 (2005-2006) and 8 (2010-2011), including 72% of the originally enrolled respondents [15] and 3,121 respondents providing survey information. In reporting this study, STROBE guidelines (STrengthening the Reporting of OBservational studies in Epidemiology) were followed [17]. For this analysis, we restricted the analytic sample to those who were nondiabetic at wave 7 (2005-2006, year 20) by excluding 318 cases of diabetes as defined by respondent self-report, anti-hyperglycemic medication use, elevated oral glucose tolerance test (OGTT: ≥200 mg/dL), elevated fasting glucose (FG: ≥126 mg/dL), or elevated glycohemoglobin values (HA1c: >6.5%) [2].

### 2.2. Measures

#### 2.2.1. Dependent Variable: Incident T2DM at Year 25 (Nondiabetic Sample in Year 20)

Given our focus on the prospective development of T2DM between wave 7 (2005-2006) and wave 8 (2010-2011), we used a 2-tiered approach to define incident T2DM. First, we used the CARDIA study participants' self-report of diabetes status and anti-hyperglycemic medication use, in combination with American Diabetes Association-defined abnormal values for the oral glucose tolerance test (OGTT: ≥200 mg/dL), fasting glucose (FG: ≥126 mg/dL), or glycohemoglobin (HA1c: >6.5%) to identify participants with the new onset of diabetes between waves 7 (2005-2006) and 8 (2010-2011). Those who do not meet these criteria were classified as nondiabetic. Next, we used the diabetes typology model (DTM) [18] to identify participants with DTM-defined likely T2DM at wave 8. DTM applied latent class analysis to six composite measures of diabetes risk. These included (a) three Homeostatic Model Assessment (HOMA) surrogates, namely, HOMA-%*β* for pancreatic *β*-cell insulin production, HOMA-IR for insulin resistance, and HOMA-%S for insulin sensitivity; (b) insulin and glucose levels; and (c) body mass index [18]. DTM identified three latent classes: likely type 1 diabetes, likely type 2 diabetes, and atypical diabetes [18]. According to DTM and for this study, the DTM defined likely-T2DM classification includes having a low prevalence of high HOMA-%S, low prevalence of low fasting insulin, high glucose to insulin ratios; low prevalence of low HOMA-IR, and low prevalence of low or normal BMI [18]. For this study, participants who presented a metabolic profile comparable to the DTM defined likely-T2DM classification were classified as likely-T2DM at wave 8 (2010-11). The DTM model was validated in a prior study using C-peptide, an endogenous marker of insulin production [18]. Validation analysis of DTM-defined likely T2DM against C-peptide among diabetics not taking insulin in the 2003-2004 NHANES data set demonstrated 75.5% sensitivity (normal/high C-peptide), 83.3% specificity (low C-peptide), and a positive predictive value of 97.42% [18]. These results demonstrated that DTM classified T2DM with a high level of certainty, and allowed us to identify study participants that were most likely to have developed T2DM in this population-based study. For these analyses, we excluded 19 cases whose T2DM status could not be determined using DTM [18] due to missing data required for the latent class analysis. The final analytic sample consisted of 2,784 participants.

#### 2.2.2. Independent Variables: Obesity and Inflammatory Biomarkers

Obesity at wave 7 was assessed using an anthropometric measure of abdominal fat: high sex-specific waist circumference (S-WC) of 35+ inches for women and 40+ for men. We used four measures of inflammatory biomarkers indicative of general or specific body inflammation [19, 20] that were assessed at wave 7. The inflammatory biomarkers used included (a) serum C-reactive protein (CRP (mg/L):<3.00 = 1;3.00‐9.99 = 2; or≥10.00 = 3), (b) high sex-specific serum uric acid (S-UA (mg/dL): <6.1=0 and ≥6.1=1 for women; <8.0=0 and ≥8.0=1 for men), (c) high serum fibrinogen (mg/dL) (0‐449 = 0and≥450 = 1), and (d) high sex-specific serum gamma-glutamyltransferase (GGT (U/L):<36 = 0and≥36 = 1for women;<61 = 0and≥61 = 1for men) [19, 21].

As used in CARDIA, race was based on self-identified Black or White race [15].

Covariates included metabolic risk factors for T2DM and diabetic dyslipidemia assessed at wave 7 [2, 22, 23]. The four metabolic risk factors included having (a) high serum triglycerides (mg/dL) (0‐199 = 0;≥200 = 1), (b) low serum sex-specific HDL cholesterol (S-HDL (mg/dL):0‐39 = 1and≥40 = 0for men;0‐49 = 1and≥50 = 0for women), (c) high total plasma cholesterol (mg/dL) (0‐199 = 0;≥200 = 1), and (d) high blood pressure (mm Hg) (≥140 *systolic* or ≥ 90 *diastolic* = 1; <140 *systolic* or < 90 *diastolic* = 0) [6].

Other wave 7 demographic covariates included sex (*woman* = 0; *man* = 1), age in years, marital status (*married* = 0; *never* *married* = 1; *divorced*/*widowed*/*separated* = 2; *living* in *a* *marriage* − *like* *relationship* = 3; or *other* = 4), employment status (*employed* = 0; *unemployed* = 1), low income status (*less* *than* $25,000 = 1; *else* = 0), smoking status (never, former, or current), and binge drinking (drank five or more alcoholic beverages on the same occasion in the past 30 days: *no* = 0; *yes* = 1). The number of first-degree family members with diabetes was assessed in wave 8 (mother, father, and/or siblings).

### 2.3. Statistical Analyses

Multivariate imputation by fully conditional specification methods was used to create 25 data sets in which each missing value was replaced by a plausible value. Data preparation and multiple imputation were performed using SAS® software version 9.4 (SAS Institute Inc., Cary, NC). Analyses were conducted on each of the 25 multiply imputed data sets, and summary estimates were calculated along with confidence intervals and *p* values using multiple imputation methods implemented in SUDAAN Release 11.0.1 (Research Triangle Institute, Research Triangle Park, NC). We used logistic regression analyses to assess the odds of developing T2DM between 2005-2006 and 2010-2011 at wave 8, testing a series of nine models: Model 1 = high sex-specific waist circumference and race; Model 2 = Model 1 plus the four metabolic risk factors (high triglycerides, low S-HDL, high total cholesterol, high blood pressure); Model 3 = Model 2 plus the four inflammatory biomarkers; and Models 1-3 stratified by race (Models 4-6 for White study participants, Models 7-9 for Black study participants). All models controlled for demographic characteristics (other than race) and behavioral risk factors. Finally, we conducted a series of mediation analyses to examine the indirect effects of waist circumference on developing T2DM through each inflammation correlate in the overall sample and by race, adjusting for demographics and metabolic and behavioral risk factors. These analyses were conducted using Mplus version 8.4 (Muthén & Muthén, Los Angeles, CA).

## 3. Results

### 3.1. Study Participant Characteristics

**Figure 1:**
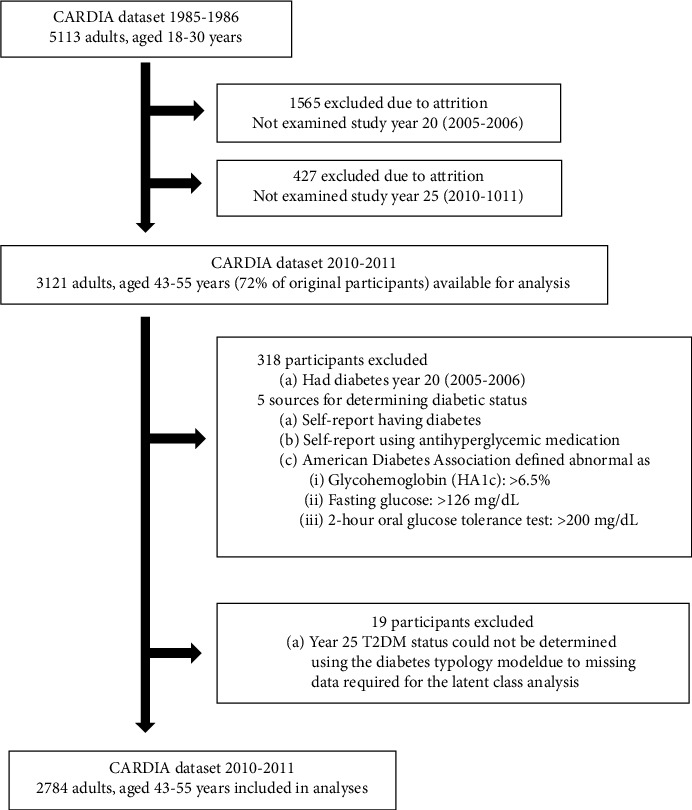
STROBE flow diagram of CARDIA study participant inclusion and exclusion criteria.

For this study, data for 3121 CARDIA participants from wave 8 of the study were screened for eligibility. A total of 2784 adults aged 43-55 in waves 7 through 8 were included in the analyses (Figure 1]. The mean age for study participants at wave 7 was 45.2 (SE 0.07) years and did not differ significantly by race. As reported by Funkhouser et al. [24], living participant retention differed by race-sex for each examination cycle; for year 25 (wave 8; 2010-2011), it was highest for White females and lowest for Black males (Table 1, *p* < 0.0001). Overall, 33.7% of the sample had high sex-specific waist circumference at wave 7 (Table 1]. Regarding the inflammatory biomarkers, `76.5% of the study participants had a *CRP* < 3.0 *mg*/*L*, 19.5% had a CRP in the 3-9.99 mg/L range, and 4.0% had a CRP that was ≥10 mg/L. 25.3% of the study participants had high fibrinogen levels, 14.3% had high sex-specific uric acid, and 12.5% had high sex-specific GGT at wave 7. One hundred and sixty-three (5.8%) participants not previously having diabetes in wave 7 developed diabetes between waves 7 (2005-2006) and 8 (2010-2011). Of those, 146 (89.6%) were algorithm-identified DTM-defined likely T2DM at wave 8. Compared to White respondents, Black respondents had a higher prevalence of high sex-specific waist circumference (42.0% vs. 27.3%; *p* < 0.0001), high C-reactive protein (25.6% vs. 14.8%; *p* < 0.0001), high fibrinogen (35.4% vs. 17.7%; *p* < 0.0001), high sex-specific uric acid (17.9% vs. 11.6%; *p* < 0.0001), and high sex-specific GGT (18.3% vs. 8.2%; *p* < 0.0001) at wave 7. Furthermore, Black respondents were more than twice as likely as White respondents to have developed T2DM at wave 8 (7.7% vs. 3.4%; *p* < 0.0001).

### 3.2. Multivariable Logistic Regression Models: Overall Sample

In Model 1 (Table 2], high sex-specific waist circumference at wave 7 was associated with higher odds of developing T2DM at wave 8 (*adjusted* *odds* *ratio* (*AOR*) = 6.15, 95%*confidence* *interval* (*CI*) = 4.14, 9.14). Blacks had higher odds of developing T2DM between waves 7 and 8 than Whites (*AOR* = 1.60, 95%*CI* = 1.05, 2.44). In Model 2, the associations of high sex-specific waist circumference and race with T2DM remained significant, with marginal attenuation of race effects when adjusting for known metabolic risk factors of T2DM. In Model 3, additionally adjusting for inflammatory biomarkers further attenuated the associations of high sex-specific waist circumference and race with T2DM, and race was no longer statistically significantly associated with T2DM. Specifically, CRP (*AOR* = 1.83, 95%*CI* = 1.18, 2.82) and high sex-specific uric acid (*AOR* = 2.57, 95%*CI* = 1.70, 3.89) were associated with T2DM, independent of metabolic risk factors and other covariates. We tested for interaction effects of race by each of the inflammatory biomarkers and race by waist circumference and found that none of these interaction effects were significant (data not shown). Additional analyses using all cases of incident diabetes, instead of DTM-defined T2DM, as the outcome variable showed that the observed associations remain significant although attenuated (data not shown). Thus, excluding cases unlikely to represent type 2 diabetes (late-onset type 1 and atypical diabetes) allows for a clearer understanding of the effects of waist circumference, race, and inflammation on the risk for developing T2DM. This is consistent with the sensitivity analyses from the original paper proposing DTM [18], which found that “compared to regression analysis on known correlates of T2DM using all diabetes cases as outcomes, using the DTM to remove likely type 1 DM and atypical DM cases resulted in a 2.5 ± 5.3%*r*-square improvement in the regression analysis, as well as model fits as indicated by significant improvement in -2 log likelihood (*p* < 0.01).”

### 3.3. Multivariable Logistic Regression Models: Race-Specific Samples

In models including obesity, while stratifying by race (Table 3], there is a significant association between sex-specific waist circumference and T2DM for both White and Black participants, with the association appearing to be stronger for Whites than Blacks (White *AOR* = 7.12, 95%*CI* = 3.77, 13.42 (Model 4) versus Black *AOR* = 5.88, 95%*CI* = 3.48, 9.93 (Model 7)). However, the race by waist circumference interaction test did not reach statistical significance (*p* = 0.55). Obesity remains significantly associated with T2DM for both Whites and Blacks when metabolic risk factors (Models 5 and 8) and inflammatory biomarkers (Models 6 and 9) are added to the models. However, there is more attenuation of the relationship between obesity and T2DM among Whites than Blacks when metabolic and inflammatory biomarkers are added to the models (Model 6). Additionally, in the full Models 6 and 9, high blood pressure was positively associated with T2DM among Whites (*AOR* = 4.42, 95%*CI* = 1.96, 9.95) but not Blacks. Male sex was positively associated with T2DM among Blacks (*AOR* = 2.07, 95%*CI* = 1.20, 3.58) but not Whites.

As shown in the full models that contain obesity, inflammatory biomarkers, and metabolic risk factors (Models 6 and 9), there is a positive association between high sex-specific serum uric acid levels and CRP with T2DM, for both Whites and Blacks. Furthermore, the associations of CRP (*AOR* = 2.59, 96%*CI* = 1.22, 5.48 for Whites versus *AOR* = 1.78, 95%*CI* = 1.04, 3.04 for Blacks) and uric acid (*AOR* = 3.48, 95%*CI* = 1.75, 6.92 for Whites versus *AOR* = 2.09, 95%*CI* = 1.24, 3.52 for Blacks) with T2DM, appeared stronger for Whites than Blacks, which suggest a higher risk for developing T2DM in Whites than Blacks given the same CRP and uric acid levels. However, race by CRP and race by uric acid interaction test failed to reach statistical significance (*p* = 0.48 and *p* = 0.18, respectively).

Finally, mediation analyses were conducted to discern if the inflammatory biomarkers explained the association between high waist circumference and T2DM. The mediation analyses, not shown, indicate that 9.9% of the association is explained by CRP and 8.1% by uric acid. Furthermore, while the direction of the association is similar for Blacks and Whites the magnitude is stronger in Whites (11.5% for uric acid, *p* < 0.001 and 13.0% for CRP 3.00-9.99 mg/L, *p* = 0.003) than Blacks (6.8% for uric acid, *p* = 0.012).

## 4. Discussion

In this prospective cohort study, we assessed the relative influence of an anthropometric marker of obesity (waist circumference), inflammatory biomarkers, and metabolic risk factors on the development of T2DM, and whether these relationships differed by race. Our results suggest important roles for waist circumference and inflammatory biomarkers CRP and uric acid, as risk factors for developing T2DM for both Blacks and Whites. The association of waist circumference and these inflammatory biomarkers with T2DM was independent of demographics, alcohol and tobacco use, and other known diabetes-related metabolic risk factors. While inflammation as measured by CRP and uric acid was an independent predictor for T2DM, inflammation as measured by the acute-phase reactant fibrinogen or the liver enzyme S-GGT was not independently associated with risk of developing T2DM in our study. Compared to Whites, nearly twice as many Black participants developed T2DM between waves 7 and 8. This racial disparity in the development of T2DM was robust to adjustments for metabolic risk factors but was fully attenuated with the inclusion of inflammatory biomarkers in the full model.

Although studies have shown a positive relationship between CRP and diabetes, this association is often reduced or attenuated completely when anthropometric body measures, such as body mass index and hip-waist ratio, are accounted for in the analyses [25]. In our study, however, we found that the association between CRP and T2DM was robust for both Blacks and Whites (Table 3], and notably stronger among Whites than Blacks even when waist circumference was added to the models. Anthropometric measures such as body mass index and waist circumference are widely used in clinical and epidemiological research on obesity; however, their relative utility as predictors of T2DM risk remains unclear. When we conducted additional analyses using obesity, defined as *BMI* ≥ 30, instead of waist circumference, we found that the association between obesity defined using *BMI* ≥ 30 and incident T2DM is weaker than that of waist circumference and T2DM, and also that race is not associated with T2DM in Models 1 and 2. We believe that this is because waist circumference is a better indicator than BMI for obesity, which is associated with developing T2DM. We do not think that we should include BMI and waist circumference in the same models because of the correlation between these two variables. Body mass index, often used as an indicator of abnormal accumulation of body fat, is limited because it does not distinguish between lean and fat mass or provide information on body fat distribution, which has been implicated in the risk for T2DM [26]. In this study, we used waist circumference rather than body mass index because it provides an estimate of abdominal fat distribution, which is more specifically associated with the development of T2DM [26]. Although CRP was associated independently with T2DM among both Blacks and Whites in our study, this relationship was stronger among Whites than Blacks, while waist circumference had a stronger association with T2DM among Blacks compared to Whites.

Multiple mechanisms have been proposed for linking GGT to T2DM including increased liver inflammation, fatty liver disease, and oxidative stress [9, 15]. However, results of observational studies evaluating the association between GGT and T2DM have been contradictory, and the utility of GGT for predicting T2DM risk remains inconclusive [9]. Similarly, although a positive association between elevated fibrinogen levels and insulin resistance has been reported [27], the relationship between hyperfibrinogenemia and insulin responsiveness remains unresolved.

Gout and renal disease are primary consequences of hyperuricemia. It has also been reported that hyperuricemia “mediates increased insulin resistance and decreased insulin release” [28], and studies have shown an association of hyperuricemia with T2DM and a wide range of health outcomes [8, 10, 28]. However, there is a lack of consensus on an appropriate threshold for hyperuricemia to be used in the assessment of T2DM risk [8]. Furthermore, an analysis of 23 meta-analyses of observational studies [29] reported that there is a lack of convincing evidence for a clear role for hyperuricemia in the development of T2DM; rather, 11 studies reported that the association between hyperuricemia and developing T2DM was highly suggestive but not convincing with incident T2DM as the outcome [8], and for 12 studies the evidence was only suggestive [30]. In our study, we identified prospectively significant associations between inflammation as indicated by increased levels of CRP and uric acid with T2DM among both Blacks and Whites.

There are some limitations to the current study. The inflammatory biomarkers we used are not comprehensive due to the unavailability of additional measures of inflammation in the CARDIA study, e.g., complete blood count and differential was only collected at baseline in 1985 and we were unable to assess the relative contribution of innate immune cells such as neutrophils, lymphocytes, and monocytes/macrophages to the development of T2DM in subsequent years. Additionally, the results for each physiologic test, including fasting glucose and the metabolic risk factors and inflammatory biomarkers, are only reflective of a single time point. While this may be consistent with how a routine clinical physical exam is performed, some measures including fasting glucose, blood pressure, and CRP are more sensitive to day-to-day fluctuations, which were not accounted for in these analyses. The study only included Black and White study participants, and may not apply to other racial groups or ethnicities. Also, the study only included middle aged adults, and results may not be applicable to other age groups. Additional studies are needed to corroborate these findings in other racial and ethnic groups, and in younger or older adults at risk for T2DM.

Although extensive data from experimental and epidemiologic investigations substantiate that low-grade systemic inflammation is a risk factor for T2DM, research efforts continue to focus on discerning the underlying disease pathophysiology, as well as identifying the most useful inflammatory biomarkers and practical ways to manage inflammation. T2DM-related inflammatory processes are dynamic, and as such, inconsistency of findings is likely reflective of shifting complex cellular responses to various stimuli that stress the pancreatic islets that produce insulin, as well as the insulin-responsive tissues including liver, muscle, and adipose tissue.

Although we did not observe definitive race-specific associations, we did observe that the associations between the inflammatory biomarkers and T2DM appeared to be stronger in Whites than in Blacks despite nonsignificant race interaction tests. Given that statistical tests for interactions are often underpowered, these findings suggest that clinical monitoring of uric acid and CRP in individuals at risk for T2DM [28] may be beneficial, and that metabolic risk factors and inflammatory biomarkers may operate differently by race related to T2DM risk. Further studies with larger sample sizes that allow for adequate comparisons by race are needed to adequately test this hypothesis. Additionally, these findings suggest that clinically targeted anti-inflammatory interventions such as using allopurinol to lower serum uric acid may reduce the risk of developing T2DM in at-risk patients [28]. Additional investigations are also needed to tease out the exact role of CRP in insulin responsiveness and aberrant glucose control as it relates to T2DM risk.

## 5. Conclusions

In conclusion, this study demonstrates that elevated levels of CRP and uric acid are positively associated with the risk of developing diabetes independent of metabolic risk as defined by elevated and or abnormal cholesterol, blood pressure, and waist circumference. These findings suggest that controlling systemic inflammation may reduce the risk of developing T2DM. Additional studies are needed to further investigate differences in the relationship between inflammatory biomarkers and diabetes by race. If our suggestive differences are confirmed, examination of inflammatory biomarker patterns of Blacks and Whites may be both beneficial and necessary when assessing patient risk for T2DM.

## Figures and Tables

**Table 1:** Study participant demographics, obesity and inflammation correlates, and behavioral and metabolic risk factors at wave 7 (2005-2006), and incident type 2 diabetes mellitus at wave 8 (2010-2011), from the Coronary Artery Risk Development in Young Adults (CARDIA) study.^a,b^.

	Full sample *n* (%)	Blacks	Whites	*p* value
*Predictors*				
High sex-specific waist circumference	937 (33.7%)	505 (42.0%)	432 (27.3%)	<0.0001
*Inflammation correlates*				
C-reactive protein (CRP, mg/L)				
<3.00	2,130 (76.5%)	811 (67.6%)	1,319 (83.3%)	<0.0001
3.00-9.99	542 (19.5%)	308 (25.6%)	235(14.8%)	
≥10.00	111 (4.0%)	82 (6.8%)	29 (1.9%)	
High fibrinogen	705 (25.3%)	425 (35.4%)	280 (17.7%)	<0.0001
High sex-specific uric acid	399 (14.3%)	216 (17.9%)	184 (11.6%)	<0.0001
High sex-specific gamma-glutamyltransferase	349 (12.5%)	219 (18.3%)	130 (8.2%)	<0.0001
*Covariates—demographics*				
*Race*				
White	1,583 (56.9%)			0.0001
Black	1,201 (43.1%)			
*Sex*				
Male	1,200 (43.1%)	460 (38.3%)	740 (46.7%)	<0.0001
Female	1,584 (56.9%)	741 (61.7%)	843 (53.3%)	<0.0001
*Age in years*	45.2 ± 0.07	44.5 ± 0.11	45.7 ± 0.08	
*Marital status*				
Married	1,581 (56.8%)	523 (43.5%)	1,059 (66.9%)	<0.0001
Never married	481 (17.3%)	284 (23.6%)	197 (12.4%)	
Divorced, separated, or widowed	380 (13.7%)	220 (18.3%)	160 (10.1%)	
Living in a marriage-like relationship	203 (7.3%)	82 (6.9%)	121 (7.7%)	
Other	138 (5.0%)	92 (7.7%)	46 (2.9%)	
*Unemployed*	314 (11.3%)	196 (16.3%)	117 (7.4%)	<0.0001
*Household income < $25,000*	400 (14.4%)	287 (23.9%)	113 (7.1%)	<0.0001
*Years of education*	14.8 ± 0.04	14.1 ± 0.05	15.4 ± 0.04	
*Immediate family members known to have diabetes in 2010-2011*	0.68 ± 0.02	0.99 ± 0.04	0.45 ± 0.02	
*Covariates*—*behavioral*				
Smoking status				<0.0001
Never	1,731 (62.2%)	752 (62.6%)	980 (61.9%)	
Former	542 (19.5%)	154 (12.8%)	388 (24.5%)	
Current	510 (18.3%)	295 (24.6%)	215 (13.6%)	
Binge drinker	594 (21.3%)	235 (19.6%)	359 (22.7%)	0.0468
*Covariates*—*metabolic risk factors*				
High triglycerides	229 (8.2%)	51 (4.2%)	178 (11.2%)	<0.0001
Low sex-specific HDL cholesterol	769 (27.6%)	330 (27.4%)	439 (27.7%)	0.8582
High total cholesterol	923 (33.1%)	393 (32.7%)	530 (33.5%)	0.6647
High blood pressure	264 (9.5%)	184 (15.3%)	80 (5.1%)	<0.0001
*Outcome*				
Likely to have T2DM in 2010-2011	146 (5.2%)	92 (7.7%)	54 (3.4%)	<0.0001

^a^Continuous variables are expressed as mean ± SEM, categorical variables are expressed as *n* (%). ^b^All independent variables and covariates except immediate family members known to have diabetes were measured in wave 7 (2005-2006).

**Table 2:** Logistic regression models of the odds of incident type 2 diabetes at wave 8 (2010-2011), CARDIA study.

	Model 1	Model 2	Model 3
*Race*	*Adjusted odds ratio * **(95% CI)**	*Adjusted odds ratio * **(95% CI)**	*Adjusted odds ratio * **(95% CI)**
Black (vs. white)	**1.60 (1.05, 2.44)**∗	**1.64 (1.06, 2.55)**∗	1.43 (0.91, 2.25)
*High sex-specific waist circumference*	**6.15 (4.14, 9.14)**∗∗∗	**5.39 (3.46, 8.40)**∗∗∗	**3.80 (2.41, 6.01)**∗∗∗
*Inflammation correlates*			
C-reactive protein (CRP, mg/L)			
3.00-9.99			**1.83 (1.18, 2.82)**∗
≥10.00			1.64 (0.81, 3.30)
High fibrinogen			1.24 (0.81, 1.92)
High sex-specific uric acid			**2.57 (1.70, 3.89)**∗∗∗
High sex-specific gamma-glutamyltransferase			0.85 (0.49, 1.47)
*Demographics*			
Age in years	1.04 (0.99, 1.09)	1.04 (0.99, 1.09)	1.03 (0.98, 1.08)
Sex (male)	1.39 (0.95, 2.02)	1.23 (0.83, 1.82)	1.47 (0.97, 2.23)
Never married	1.15 (0.70, 1.90)	1.18 (0.71, 1.96)	1.16 (0.69, 1.93)
Divorced/widow/separated	1.06 (0.63, 1.80)	1.05 (0.62, 1.78)	1.04 (0.59, 1.81)
Living in a marriage-like relationship	0.55 (0.22, 1.36)	0.52 (0.20, 1.32)	0.53 (0.20, 1.43)
Other marital status	1.07 (0.50, 2.29)	1.09 (0.50, 2.34)	1.20 (0.54, 2.67)
Unemployed	1.03 (0.59, 1.78)	1.00 (0.57, 1.75)	1.03 (0.58, 1.83)
Household income < $25,000	0.62 (0.36, 1.08)	0.60 (0.35, 1.06)	0.60 (0.34, 1.06)
Years of education	0.94 (0.85, 1.04)	0.95 (0.86, 1.05)	0.95 (0.86, 1.05)
Immediate family members known to have DM	**1.33 (1.16, 1.52)**∗∗	**1.37 (1.19, 1.57)**∗∗∗	**1.35 (1.17, 1.55)**∗∗∗
*Behavioral factors*			
Smoking status			
Former	1.33 (0.84, 2.10)	1.37 (0.86, 2.19)	1.48 (0.92, 2.38)
Current	1.44 (0.90, 2.30)	1.36 (0.85, 2.20)	1.39 (0.85, 2.27)
Binge drinker	0.64 (0.38, 1.07)	0.63 (0.37, 1.05)	0.60 (0.35, 1.04)
*Metabolic risk factors*			
High triglycerides		**2.03 (1.14, 3.62)**∗	**1.99 (1.08, 3.67)**∗
Low sex-specific HDL cholesterol		1.00 (0.68, 1.49)	0.90 (0.60,1.34)
High total cholesterol		1.10 (0.76, 1.61)	1.06 (0.72, 1.55)
High blood pressure		**1.93 (1.21, 3.07)**∗	**1.90 (1.17, 3.11)**∗

^∗∗∗^ *p* < 0.0001; ^∗∗^*p* < 0.001; ^∗^*p* < 0.05.

**Table 3:** Logistic regression models of the odds of incident type 2 diabetes at wave 8 (2010-2011), by race (CARDIA study).

	Model 4	Model 5	Model 6	Model 7	Model 8	Model 9
	White			Black		
	Adjusted odds ratio (95% CI)			Adjusted odds ratio (95% CI)		
*High sex-specific waist circumference*	**7.12 (3.77, 13.42)**∗∗∗	**4.77 (2.30, 9.86)**∗∗∗	**3.31 (1.50, 7.26)**∗∗	**5.88 (3.48, 9.93)**∗∗∗	**5.94 (3.35, 10.53)**∗∗∗	**4.09 (2.29, 7.29)**∗∗∗
*Inflammation correlates*						
C-reactive protein (CRP, mg/L)						
3.00-9.99			**2.59 (1.22, 5.48)**∗			**1.78 (1.04, 3.04)**∗
≥10.00			1.13 (0.16, 7.79)			2.01 (0.92, 4.42)
High sex-specific uric acid			**3.48 (1.75, 6.92)**∗∗			**2.09 (1.24, 3.52)**∗
High fibrinogen			0.76 (0.33, 1.72)			1.51 (0.89, 2.57)
High sex-specific gamma-glutamyltransferase			0.67 (0.23, 1.94)			0.86 (0.45, 1.65)
*Demographics*						
Age in years	1.00 (0.91, 1.09)	1.01 (0.92, 1.12)	1.02 (0.91, 1.13)	**1.06 (1.00, 1.12)**∗	1.05 (0.99, 1.11)	1.03 (0.97, 1.10)
Sex (male)	1.13 (0.63, 2.02)	0.89 (0.48, 1.64)	1.10 (0.58, 2.09)	**1.68 (1.02, 2.74)**∗	1.61 (0.97, 2.67)	**2.07 (1.20, 3.58)**∗
Never married	1.20 (0.49, 2.93)	1.28 (0.52, 3.14)	1.19 (0.44, 3.22)	1.20 (0.64, 2.23)	1.19 (0.64, 2.22)	1.16 (0.62, 2.16)
Divorced/widowed/separated	1.29 (0.54, 3.11)	1.17 (0.47, 2.87)	1.14 (0.40, 3.26)	0.93 (0.48, 1.80)	0.95 (0.49, 1.84)	0.97 (0.49, 1.91)
Living in a marriage-like relationship	0.48 (0.10, 2.25)	0.44 (0.09, 2.10)	0.44 (0.07, 2.58)	0.60 (0.19, 1.83)	0.58 (0.19, 1.82)	0.59 (0.18, 1.97)
Other marital status	0.72 (0.10, 5.13)	0.89 (0.12, 6.36)	1.06 (0.15, 7.56)	1.22 (0.51, 2.94)	1.22 (0.50, 2.96)	1.37 (0.54, 3.45)
Unemployed	1.32 (0.43, 4.05)	1.19 (0.38, 3.75)	1.23 (0.38, 4.03)	0.90 (0.47, 1.75)	0.88 (0.45, 1.72)	0.87 (0.44, 1.72)
Household income < $25,000	0.38 (0.09, 1.63)	0.37 (0.09, 1.58)	0.40 (0.10, 1.68)	0.69 (0.37, 1.28)	0.69 (0.37, 1.28)	0.67 (0.35, 1.26)
Years of education	0.94 (0.80, 1.11)	0.98 (0.82, 1.16)	1.00 (0.83, 1.20)	0.96 (0.85, 1.08)	0.96 (0.84, 1.08)	0.95 (0.83, 1.08)
Immediate family members known to have diabetes	**1.93 (1.44, 2.60)**∗∗∗	**2.02 (1.50, 2.71)**∗∗∗	**2.03 (1.50, 2.76)**∗∗∗	**1.19 (1.03, 1.38)**∗	**1.24 (1.06, 1.44)**∗∗	**1.22 (1.05, 1.41)**∗
*Behavioral*						
Smoking status						
Former	1.16 (0.59, 2.26)	1.39 (0.70, 2.76)	1.46 (0.72, 2.97)	1.45 (0.77, 2.71)	1.51 (0.80, 2.84)	1.73 (0.91, 3.29)
Current	1.41 (0.61, 3.23)	1.28 (0.54, 3.06)	1.35 (0.55, 3.32)	1.40 (0.78, 2.50)	1.39 (0.77, 2.51)	1.41 (0.77, 2.61)
Binge drinker	0.59 (0.26, 1.32)	0.59 (0.25, 1.41)	0.55 (0.22, 1.33)	0.67 (0.33, 1.33)	0.62 (0.31, 1.24)	0.62 (0.30, 1.29)
*Metabolic risk factors*						
High triglycerides		1.92 (0.88, 4.18)	1.85 (0.78, 4.39)		2.05 (0.79, 5.30)	2.12 (0.78, 5.78)
Low sex-specific HDL cholesterol		1.82 (0.92, 3.61)	1.56 (0.76, 3.18)		0.73 (0.43, 1.23)	0.67 (0.39, 1.14)
High total cholesterol		0.80 (0.44, 1.45)	0.81 (0.44, 1.52)		1.30 (0.80, 2.11)	1.27 (0.78, 2.08)
High blood pressure		**3.90 (1.81, 8.38)**∗∗	**4.42 (1.96, 9.95)**∗∗		1.34 (0.76, 2.38)	1.30 (0.71, 2.35)

^∗∗∗^ *p* < 0.0001; ^∗∗^*p* < 0.001; ^∗^*p* < 0.05.

## Data Availability

CARDIA data are available on the NHLBI website for researchers upon request. The material transfer agreement signed between NHLBI and NIMHD prohibits the authors from releasing or distributing research data in any form to any entity or individual. The data can be accessed/requested at the following link: https://biolincc.nhlbi.nih.gov/home/.

## Abbreviations

T2DM: Type 2 diabetes mellitus
DTM: Diabetes typology model
AOR: Adjusted odds ratio
CI: Confidence interval
OGTT: Oral glucose tolerance test
FG: Fasting glucose
HA1c: Glycohemoglobin
HOMA: Homeostatic model assessment
CRP: C-reactive protein
GGT: Gamma-glutamyltransferase
HDL: HDL cholesterol.
